# The transcription factor PHR1 plays a key role in the regulation of sulfate shoot-to-root flux upon phosphate starvation in Arabidopsis

**DOI:** 10.1186/1471-2229-11-19

**Published:** 2011-01-24

**Authors:** Hatem Rouached, David Secco, Bulak Arpat, Yves Poirier

**Affiliations:** 1 Department of Plant Molecular Biology, University of Lausanne, CH-1015 Lausanne, Switzerland

## Abstract

**Background:**

Sulfate and phosphate are both vital macronutrients required for plant growth and development. Despite evidence for interaction between sulfate and phosphate homeostasis, no transcriptional factor has yet been identified in higher plants that affects, at the gene expression and physiological levels, the response to both elements. This work was aimed at examining whether PHR1, a transcription factor previously shown to participate in the regulation of genes involved in phosphate homeostasis, also contributed to the regulation and activity of genes involved in sulfate inter-organ transport.

**Results:**

Among the genes implicated in sulfate transport in *Arabidopsis thaliana*, *SULTR1;3 *and *SULTR3;4 *showed up-regulation of transcripts in plants grown under phosphate-deficient conditions. The promoter of *SULTR1;3 *contains a motif that is potentially recognizable by PHR1. Using the *phr1 *mutant, we showed that *SULTR1;3 *up-regulation following phosphate deficiency was dependent on PHR1. Furthermore, transcript up-regulation was found in phosphate-deficient shoots of the *phr1 *mutant for *SULTR2;1 *and *SULTR3;4*, indicating that PHR1 played both a positive and negative role on the expression of genes encoding sulfate transporters. Importantly, both *phr1 *and *sultr1;3 *mutants displayed a reduction in their sulfate shoot-to-root transfer capacity compared to wild-type plants under phosphate-deficient conditions.

**Conclusions:**

This study reveals that PHR1 plays an important role in sulfate inter-organ transport, in particular on the regulation of the *SULTR1;3 *gene and its impact on shoot-to-root sulfate transport in phosphate-deficient plants. PHR1 thus contributes to the homeostasis of both sulfate and phosphate in plants under phosphate deficiency. Such a function is also conserved in *Chlamydomonas reinhardtii *via the PHR1 ortholog PSR1.

## Background

Sulfur and phosphorus are two of the most important macro-elements for plant growth. Given their vital roles in sustaining growth, and their participation in related metabolic pathways, plants have evolved coordinated and tightly controlled mechanisms to maintain intracellular sulfur and phosphorus homeostasis in response to varying levels of external element availability. One example of their interdependency is the rapid replacement of sulfolipids by phospholipids under sulfur deficiency, and the replacement of phospholipids by sulfolipids during phosphorus deficiency [1-4]. The responses of plants to phosphorus and sulfur deficiency have largely been examined considering each element separately; however, the interaction and crosstalk between sulfur and phosphorus signaling pathways has been poorly studied [5,6].

In plants, sulfur is acquired from the soil in its inorganic form of sulfate by the root system [7,8]. A major portion of the absorbed sulfate is transported into the vacuole and the remaining portion is loaded into the xylem and then transferred to the shoots [9]. In leaves, sulfate is reduced in the chloroplast and then assimilated into organic sulfur compounds, such as methionine, cysteine and glutathione. Transport of sulfate is mediated by members of the *SULTR *gene family containing 12 members in *Arabidopsis thaliana *that are subdivided into four groups. Members of group 1 encode high-affinity sulfate transporters, such as SULTR1;1 and SULTR1;2, that are involved in sulfate uptake into the root [10,11]. Sulfate limitation also involves redistribution of sulfate from source to sink organs through the phloem vessels, a process mediated by the phloem-localized high-affinity sulfate transporter SULTR1;3 [12]. Group 2 encode low-affinity sulfate transporters and includes SULTR2;1, which is expressed in the xylem parenchyma and pericycle cells of roots and strongly up-regulated by sulfate deficiency [13]. Group 3 is the largest group of sulfate transporters with five members. SULTR3;5 functions in synergy with SULTR2;1 in mediating low-affinity sulfate transport when expressed in yeast and participates in root-to-shoot sulfate transport [14]. A role for several SULTR3 members, including SULTR3;4, in sulfate translocation within developing seeds has also been recently described [15]. Group 4 contains only two members. SULTR4;1 and SUTR4;2 are localized to the tonoplast and are proposed to play a role in the efflux of sulfate into the cytoplasm [16]. Seeds of the *sultr4;1 *mutant have an enhanced sulfate content [17]. The role of the two members of the *SULTR5 *gene family in sulfate metabolism is uncertain, as *SULTR5;2 *has only been demonstrated to encode a high-affinity molybdate transporter [18].

At present, relatively little is known about transcription factors that participate in the control of sulfate transporters under sulfate deficiency [19,20]. In Arabidopsis, only one gene encoding the transcription factor Sulfur Limitation 1 (SLIM1) has been shown to play a role in the regulation of the expression of several sulfate transporters, such as *SULTR1;1*, *SULTR1;2 *and *SULTR4;2 *[21]. Sulfate limitation also induces the expression of microRNA miR395 in a SLIM1-dependent manner [22,23]. In turn, mirR395 regulates the accumulation and allocation of sulfate through the targeting of members of the ATP sulfurylase gene family (*APS1*, *APS3 *and *APS4*) and the *SULTR2;1 *gene [24].

Some transcription factors participating in the response of plant to inorganic phosphate (Pi) deficiency have been identified, including PHR1 [25], WRKY75 [26], ZAT6 [27] and MYB62 [28]. The PHR1 transcription factor is viewed as a positive regulator of Pi starvation responses and is involved in the up-regulation of the *IPS1 *gene in Pi-deficient plants [25]. In the promoter of *IPS1*, PHR1 binds the P1BS *cis*-acting element, defined as the imperfect palindromic sequence GNATATNC [25]. Similar palindromic sequences have been identified in the promoter region of several genes induced by Pi deficiency and positively regulated by PHR1, including the Pi transporter *PHT1;1*, *PHO1;H1 *involved in root-to-shoot Pi transfer, and the *SQD1 *and *DGD2 *genes involved in sulfolipid and galactolipid biosynthesis, respectively [29,30]. The *phr1 *mutant shows impairment in a broad range of Pi-deficiency responses, including decreased accumulation of anthocyanin, starch and sugars, altered Pi allocation between root and shoot, and decreased response of Pi starvation-induced genes [25,31]. PHR1 has also been shown to influence the expression of microRNA miR399, and forms, along with PHO2, an important branch in the long-distance Pi signaling pathway [25,29,32-35]. While *PHR1 *expression is not regulated by Pi status [25], the protein is sumoylated by the SUMO E3 ligase SIZ1, revealing a possible post-translational mechanism for PHR1 regulation [36]. Several microarray studies revealed that the promoter region of genes up-regulated by Pi deficiency, including genes systemically controlled by low Pi, are particularly enriched for the P1BS element relative to non-induced genes [37-40]. Analysis of gene expression profile and phenotypes of the *phr1 *mutant and a *phr1 phr1-like *(*phl1*) double mutant, combined with overexpression of PHR1, revealed that PHR1 and PHL1 act as central integrators of the Pi starvation response in Arabidopsis [37].

Fine tuning of the crosstalk between the regulation of phosphorus and sulfur homeostasis, both at the transcriptional and metabolic level, has been demonstrated in the unicellular alga *Chlamydomonas reinhardtii *[41] and in *Saccharomyces cerevisiae *[42,43]. However, in higher plants, although evidence suggests a similar coordination between phosphorus and sulfur homeostasis, the molecular mechanisms that regulate the sulfate homeostasis in response to Pi availability remain largely unknown. Using bioinformatics analysis, we found that the P1BS *cis*-acting element was present in the promoters of the genes *SULTR1;3 *and *SULTR2;1*, raising the possibility of the involvement of PHR1 in the crosstalk between sulfate and Pi signaling pathways in Arabidopsis. We thus first studied the transcriptional regulation of these two genes, as well as of *SULTR3;5 *and *SULTR3;4*, in wild-type (WT) Arabidopsis and *phr1 *mutant grown on Pi-depleted medium. Our results showed that *SULTR *genes were differentially regulated at the transcriptional level by the Pi status in plants and that PHR1 could play a positive role in the expression of *SULTR1;3*, and a negative role in the expression of *SULTR2;1 *and *SULTR3;4*. Under Pi-deficient conditions, the sulfate shoot-to-root transfer capacity of the *phr1 *and *sultr1;3 *mutants was reduced compared to WT and the *sultr2;1 *mutants. These results showed that the transcription factor PHR1 up-regulated the expression of *SULTR1;3*, and exercised a positive control on sulfate inter-organ distribution mediated by SULTR1;3 upon Pi starvation.

## Results

### Pi starvation alters the expression of the sulfate transporter genes

**Figure 1:**
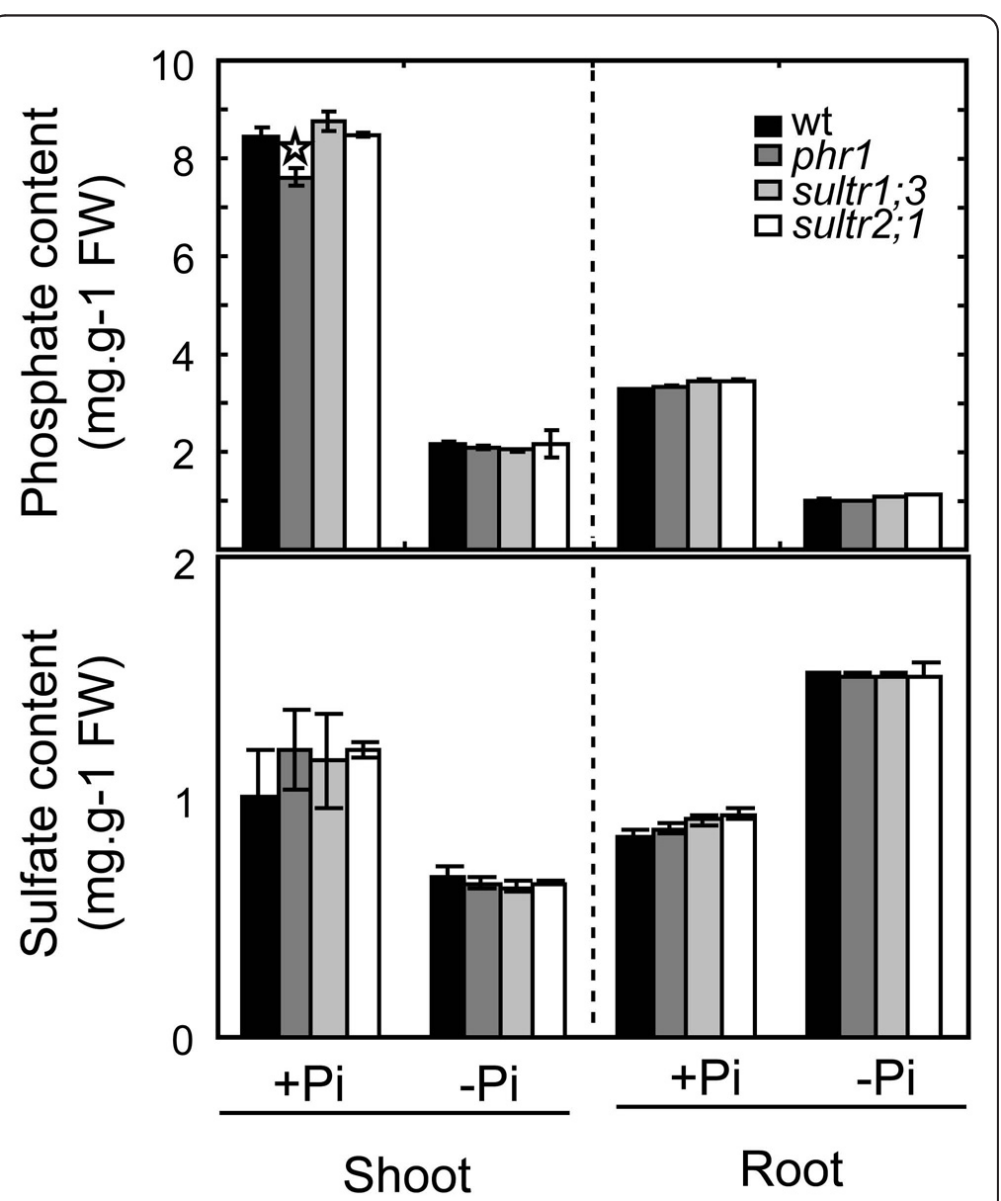
**Effect of Pi availability on the sulfate and Pi contents in Arabidopsis tissues**. Wild-type (wt) plants as well as the *phr1*, *sultr1;3 *and *sultr2;1 *mutant plants were grown on medium containing 1 mM Pi for 7 days, followed by an additional 4 days in media containing either 1 mM Pi (+Pi) or no Pi (-Pi). Shoots and roots were harvested separately and Pi (upper histograms) and sulfate (lower histograms) concentrations were quantified using high-pressure ionic chromatography. Individual measurements were obtained from the analysis of shoots or roots collected from a pool of 'n' plants (n ≥ 10). Error bars indicate SD; biological repeats (n ≥ 3). The star indicates a significant difference with WT plants (ANOVA and Tukey test, P < 0.05).

In order to determine the effect of Pi deficiency on sulfate distribution in Arabidopsis, plants were grown in medium with high Pi (1 mM) for 7 days followed by growth in medium with low Pi (10 μM) for an additional 4 days. Shoots and roots were collected separately and Pi and sulfate contents were determined. As expected, Pi starvation led to a decrease in Pi content in shoots and roots (Figure 1). In contrast, although the sulfate concentration increased 1.8-fold in roots, it decreased 1.4-fold in shoots (Figure 1). This result implies the existence of a process modifying plant root to shoot sulfate distribution under Pi deficiency.

**Figure 2:**
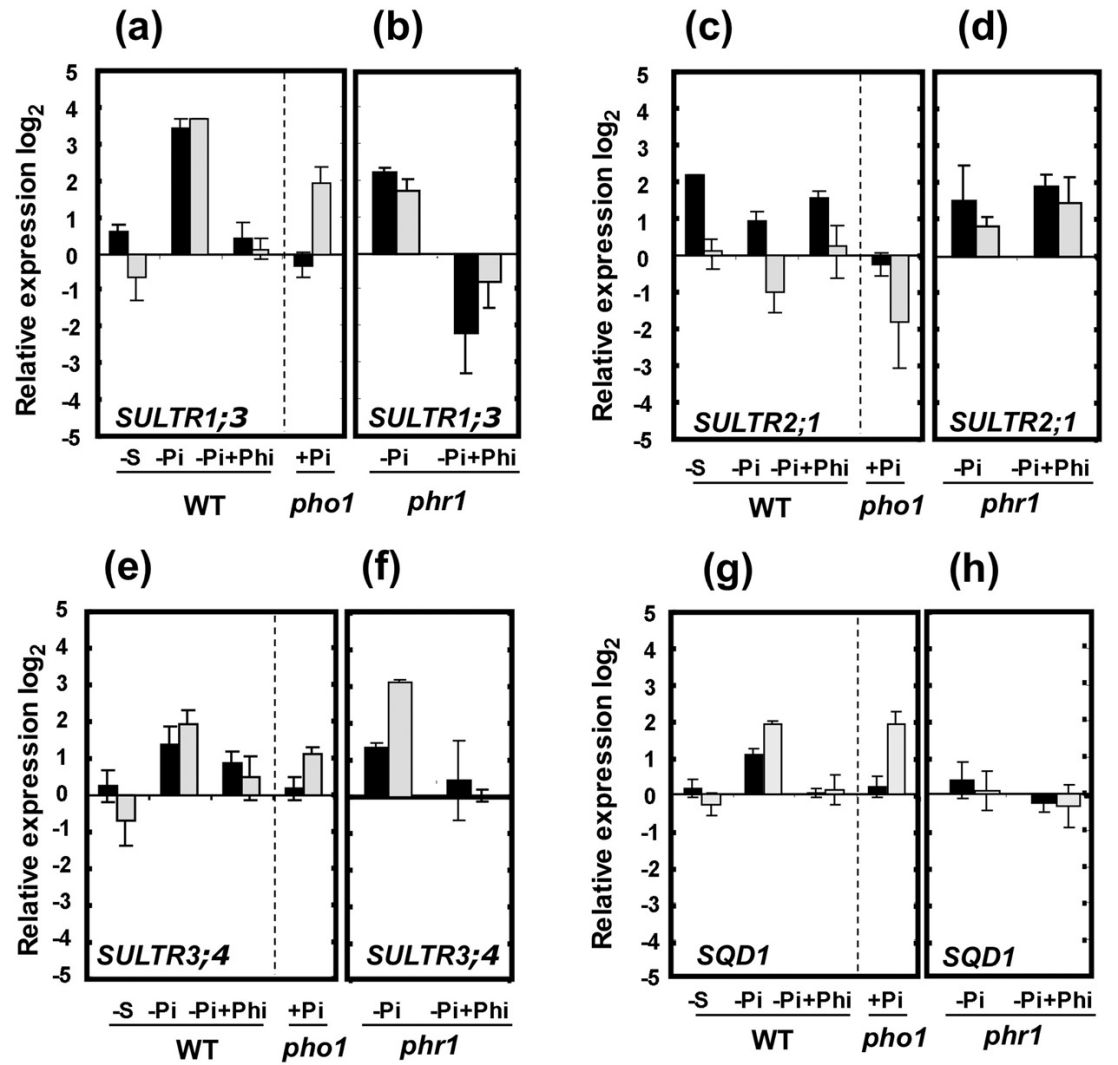
***SULTR1;3*, *SULTR2;1 *and *SULTR3;4 *mRNA accumulation in response to Pi and sulfate availability**. For Pi treatments, WT and *phr1 *mutant plants were grown on medium containing 1 mM Pi for 7 days, followed by an additional 4 days in media containing 1 mM Pi (+Pi), no Pi (-Pi) or 1 mM phosphite (+Phi). For sulfate treatments, plants were grown on medium containing 1 mM sulfate for 7 d, followed by an additional 4 days on sulfate-free medium (-S). The *pho1 *mutant was grown on complete medium containing 1 mM Pi and 1 mM sulfate for 11 days. Shoots and roots were separately harvested and mRNA accumulation was quantified by Q-RT-PCR. mRNA abundance of *SULTR1;3 *(a, b), *SULTR2;1 *(c, d), *SULTR3;4 *(e, f) and *SQD1 *(g, h) for all genotypes and treatments was normalized to the level of the control gene ubiquitin mRNA (UBQ10: At4g05320) and expressed as relative values against WT plants grown in complete (+Pi and +sulfate) medium. Expression level is expressed as log_2 _values. Black and gray histograms represent values for roots and shoots, respectively. Individual measurements were obtained from the analysis of shoots or roots collected from a pool of 'n' plants (n ≥ 12). Error bars indicate SD; biological repeats (n ≥ 3).

In Arabidopsis, three proteins have been implicated in long-distance sulfate transport between the roots and shoots. SULTR1;3 is involved in the transfer of sulfate from shoot to root [12], and SULTR3;5 modulates sulfate transport from root to shoot, potentially via its cooperation with SULTR2;1 [14]. Transcript abundance was thus first determined by quantitative RT-PCR for these corresponding genes in shoots and roots of plants grown in media with low and high Pi or sulfate (Figure 2a, c, g). Expression of the *SQD1 *gene, involved in sulfolipid biosynthesis, was also included as a control, since this gene has been previously reported to be up-regulated by Pi deficiency [1,25,30]. The *SULTR1;3 *transcript was strongly increased in both roots and shoots of Pi-deficient plants, while it was only weakly induced in roots of sulfate-deficient plants (Figure 2a). Transcript abundance of *SULTR2;1 *showed a weak increase only in roots under Pi deprivation, and a moderate increase in roots under sulfate deprivation (Figure 2c). There was no increase in *SULTR3;5 *expression under both Pi and sulfate deficiency (data not shown), while *SQD1 *expression was unchanged under sulfate deficiency but increased in both shoots and roots under Pi deficiency (Figure 2g).

To explore whether expression of any other member of the *SULTR *gene family was increased by Pi deficiency, microarray data previously generated from Pi-deficient plants were first analyzed [38,39,44,45]. Only *SULTR3;4 *was consistently induced in Pi-deficient plants in these studies. Analysis by Q-RT-PCR showed that *SULTR3;4 *expression increased moderately under Pi deficiency in both roots and shoots, while there was no change in expression under sulfate deficiency (Figure 2e).

Gene expression was examined in the *pho1 *mutant (deficient in the transfer of Pi from roots to shoots [46]) to investigate whether the increase in transcript levels under Pi deficiency could be controlled by the local level of intracellular Pi as opposed to a systemic response to Pi status emanating from the roots. When grown in complete medium, *pho1 *mutant shoots are Pi-deficient but roots are Pi-sufficient [46]. While expression of *SULTR1;3*, *SULTR2;1*, *SULTR3;4 *and *SQD1 *in roots of *pho1 *plants was not induced, shoots still over-expressed *SULTR1;3*, *SULTR3;4 *and *SQD1 *(Figure 2a, c, e, g). These results indicate that the expression of *SULTR1;3*, *SULTR3;4 *and *SQD1 *was regulated at least partially by the local tissue Pi content instead of a systemic signal initiated in the roots.

Transcript levels were further examined in plants grown in Pi-deficient medium supplemented with 1 mM phosphite. Phosphite is a reduced analogue of Pi that is readily absorbed but neither oxidized nor metabolized by plants. Studies in several plants have shown that numerous molecular and developmental responses to Pi limitations are repressed by phosphite, indicating that phosphite interferes specifically with early events involved in Pi sensing and signaling, including responses typically associated with local Pi sensing or long-distance signaling [47-50]. While addition of phosphite attenuated the induction of *SULTR1;3*, *SULTR3;4 *and *SQD1 *by Pi deficiency in shoot and roots, the same treatment did not lead to decreased *SULTR2;1 *expression (Figure 2a, c, e, g).

### PHR1 regulates the expression of *SULTR1;3*, *SULTR2;1 *and *SULTR3;4*

**Figure 3:**
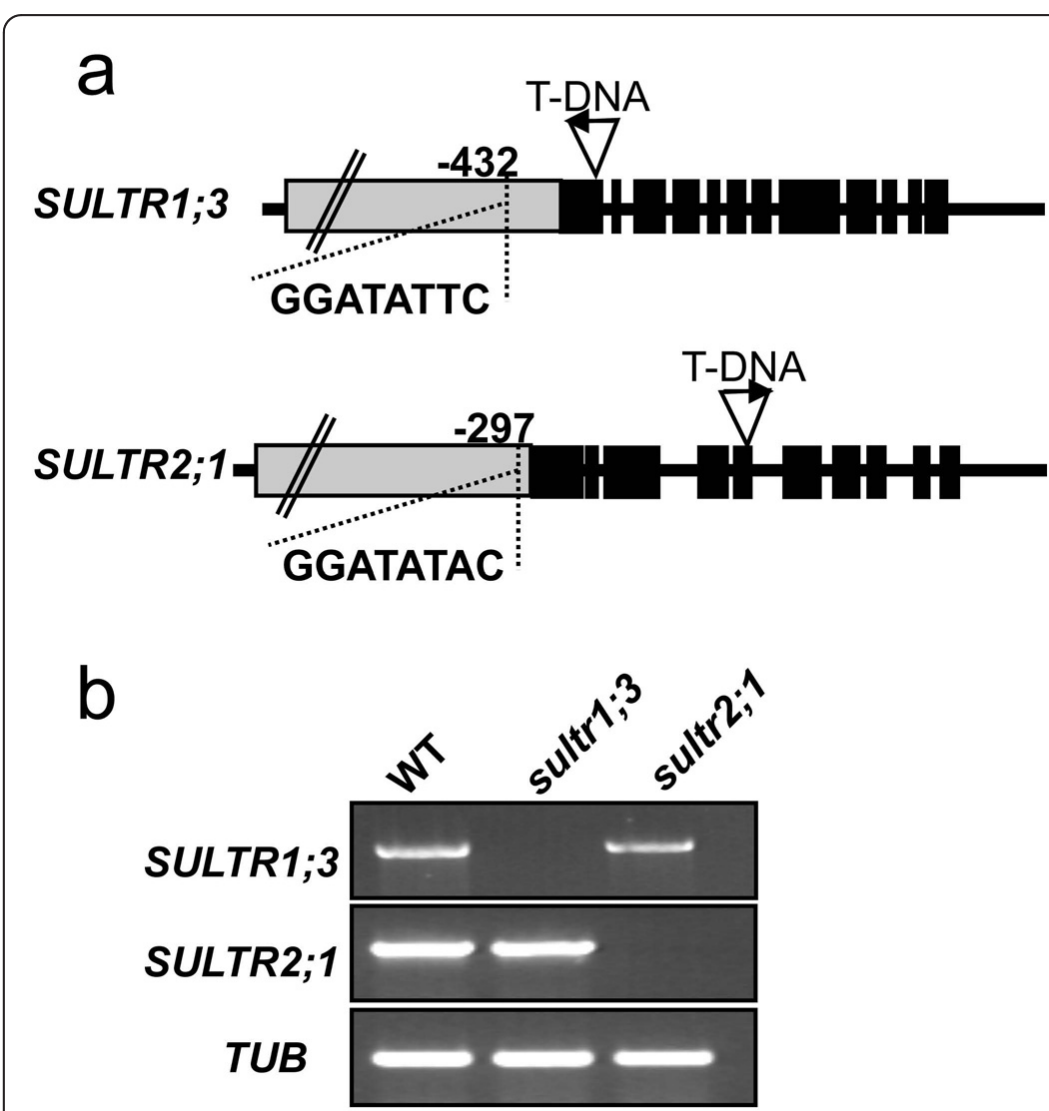
**Isolation of T-DNA mutants in the *SULTR1;3 *and *SULTR2;1 *genes**. (a) Gene structure of *SULTR1;3 *and *SULTR2;1*. Gray boxes represent promoter regions, black boxes represent exons, and lines represent introns (not drawn to scale). Insertion sites of T-DNA and orientation (from left to right borders) are represented by triangles with arrowheads. Positions (base pairs from the start codon), and sequences analogous to the PHR1-binding motif present in the promoter are indicated. (b) The absence of *SULTR1;3 *and *SULTR2;1 *transcripts in the *sultr1;3 *and *sultr2;1 *mutants, respectively, was confirmed by RT-PCR. The expression of tubulin (*TUB*) was used as a control.

Among the genes involved in sulfur metabolism, only *SQD1 *and *SQD2*, involved in sulfolipid biosynthesis, have been reported to contain the PHR1-binding motif P1BS (GNATATNC) within their promoter [25,29]. Analysis of the Arabidopsis genome for the P1BS motif within the 500-bp 5'-upstream regulatory sequences identified 3305 genes predicted to contain at least one putative PHR1-binding site. Among this set, only the *SULTR2;1 *and *SULTR1;3 *genes were identified as additional genes involved in sulfur metabolism that contained a sequence similar to the P1BS motif. The motifs GGATATTC and GGATATAC are found 432 and 297 bp upstream of the start codon of the *SULTR1;3 *and *SULTR2;1 *genes, respectively (Figure 3a). Although induction of *SULTR1;3 *in Pi-deficient roots and shoots still occurred in the *phr1 *mutant, it was strongly reduced in both tissues compared to WT plants (Figure 2a, b). A similar level of attenuation without complete loss of induction by Pi deficiency has also been observed for *IPS1 *and several other genes containing a P1BS sequence and is likely explained by the presence of a functional homolog of *PHR1 *named *PHR1-like *(*PHL*) [25,37]. As in the case of WT plants, addition of phosphite to Pi-deficient *phr1 *mutant led to the absence of induction of *SULTR1;3 *under Pi deficiency (Figure 2b). In contrast to *SULTR1;3*, the *SULTR2;1 *expression was higher, particularly in shoots, of Pi-deficient *phr1 *plants as compared to WT plants, when treated with or without phosphite (Figure 2c, d). A further increase in *SULTR3;4 *expression in the Pi-deficient *phr1 *mutant compared to WT was also observed in shoots (Figure 2e, f). However, as observed in WT plants, addition of phosphite abolished any induction of *SULTR3;4 *by Pi deficiency in *phr1 *(Figure 2f). There was strong attenuation of *SQD1 *expression in Pi-deficient plants in the *phr1 *mutant, both with and without phosphite (Figure 2g, h).

Although the Pi content in shoots of the *phr1 *mutant was slightly lower than WT for plants grown under Pi-sufficient conditions, consistent with the study of Rubio *et al. *[25], there was no significant difference between WT and *phr1 *in the contents of sulfate or Pi for plants grown under Pi-deficient conditions (Figure 1). These results indicate that the changes in gene expression in the *phr1 *mutant under Pi-deficient conditions were not due to changes in Pi or sulfate levels in the *phr1 *mutant compared to WT. Altogether, these results reveal that PHR1 had a positive effect on the expression of *SULTR1;3 *upon Pi deficiency, but a negative effect on the expression of *SULTR2;1 *and *SULTR3;4*.

### PHR1 contributes to shoot-to-root sulfate transport

A potential functional role of PHR1 in sulfate homeostasis was assessed by examining the root-to-shoot and shoot-to-root sulfate transfer in the *phr1 *mutant in comparison to the *sultr1;3 *and *sultr2;1 *single mutants and WT plants. T-DNA insertion mutants were isolated for the *SULTR1;3 *and *SULTR2;1 *genes (Figure 3a), and the absence of gene expression in homozygous mutants was confirmed by RT-PCR (Figure 3b). There were no notable differences in growth in fertilized soil of the *sultr1;3 *and *sultr2;1 *mutants in comparison to *phr1 *mutant or WT plants (data not shown).

The amount of sulfate and phosphate in the shoots and roots of the *sultr1;3 *and *sultr2;1 *mutants were not significantly different from WT, for plants grown under Pi-sufficient or Pi-deficient conditions (Figure 1). The capacity of the mutant lines to transfer radiolabeled sulfate (^35^S) from roots to shoots and vice-versa was determined for plants grown in media with high or low Pi (Table 1). There were no significant differences in the root-to-shoot sulfate transfer among all genotypes tested, for both Pi treatments. The movement of ^35^S-labeled sulfate from the shoot to root was not significantly different between WT and the various mutants under Pi-sufficient conditions; however, there was a significant reduction under Pi-deficiency for both *phr1 *and *sultr1;3 *mutants compared to Pi-deficient WT and the *sultr2;1 *mutant (Table 1).

**Table 1:** Bidirectional movement of ^35^S-labeled sulfate in wild-type, *phr1*, *sultr1;3 *and *sultr2;1*.

**Genotypes **^**a**^	^**35**^**S transfer **^**b**^			
	
	Root-to-Shoot		Shoot-to-Root	
	
	+Pi	-Pi	+Pi	-Pi
Wild-type (Col-0)	31.05 ± 1.07	33.23 ± 4.89	1.33 ± 0.85	1.51 ± 0.27

*phr1*	27.93 ± 3.10	29.73 ± 5.51	0.95 ± 0.34	0.83 ± 0.12*

*sultr1;3*	29.08 ± 1.96	32.45 ± 5.72	1.16 ± 0.23	0.63 ± 0.28*

*sultr2;1*	29.64 ± 2.46	33.36 ± 0.53	1.03 ± 0.25	1.42 ± 0.42

^a ^The treatments are described in 'Materials and Methods'.

^b ^Radioactivity transfers to the distal organs were detected after 90 min of incubation. Values are expressed as % of total radioactivity. Average ± SD (*n *= 4) was determined in three independent experiments.

* Statistical analysis performed by ANOVA reveals significant difference with wild-type -Pi treatment (P < 0.005).

Statistical analysis was performed using JMP 7 software.

## Discussion

Pi plays a central role in numerous aspects of plant metabolism, and Pi deficiency has profound effects on numerous metabolic pathways as well as on gene expression [38,39,44,51]. These changes in gene expression are expected to help plants adapt to Pi deprivation and adjust their metabolism to sustain growth and ensure survival. Several of the metabolic adjustments triggered by Pi deficiency are expected to have a direct effect on sulfate acquisition and use; e.g. Pi deficiency leads to the replacement of phospholipids by sulfolipid and galactolipids, as well to an increase in glutathione level [1,52]. Although it is expected that a certain level of coordination and crosstalk must exist between pathways involved in Pi and sulfate transport and homeostasis in plants, important players acting in this coordination remain to be clearly identified.

This current work shows that *SULTR1;3*, *SULTR2;1 *and *SULTR3;4 *genes are up-regulated by Pi deficiency. The pattern of expression of these genes in the *pho1 *mutant, with basal level of expression in Pi-sufficient roots but overexpression in Pi-deficient shoots, suggests that the response of these genes was mainly associated with a local perception of Pi deficiency. A similar pattern of expression for several Pi deficiency-responsive genes in the *pho1 *mutant was recently described [53]. Induction of *SULTR1;3 *and *SULTR3;4*, by Pi deficiency was suppressed by the Pi analogue phosphite. Studies in several plants have shown that numerous molecular and developmental responses to Pi limitations are repressed by phosphite, indicating that phosphite interferes specifically with early events involved in Pi sensing and signaling, including responses typically associated with local Pi sensing or long-distance signaling [47-50]. The influence of phosphite on *SULTR1;3 *and *SULTR3;4 *expression during Pi deficiency places these genes under the influence of this major Pi signal-transduction pathway. However, genes have also been previously identified which are induced by Pi starvation but not suppressed by phosphite, such as the Arabidopsis *PHO1 *and *PHO1;H10 *[30,54], indicating the presence of several distinct Pi deficiency signal-transduction pathways, including phosphite-sensitive and phosphite-insensitive pathways. Our data show that *SULTR2;1 *belongs to this group of Pi-inducible genes not responding to phosphite.

The present study identified an important role for both SULTR1;3 and PHR1 in the regulation of sulfate inter-organ flux upon Pi starvation in Arabidopsis. PHR1 is a transcription factor that positively regulates the expression of numerous genes upon Pi deficiency and that forms, along with *PHO2 *and the microRNA miR399, an important branch in the long-distance Pi signaling pathway [25,29,32-35]. SULTR1;3 has previously been identified as a high-affinity sulfate transporter expressed in sieve-element-companion-cell complexes of the phloem in cotyledons and roots and involved in sulfate transport from source to sink [12]. Yoshimoto *et al. *[12] revealed a small but significant decrease in shoot-to-root sulfate transport in the *sultr1;3 *mutant under nutrient sufficient conditions; however, in our experiment there was no such significant difference under Pi-sufficient conditions. The experiments reported by Yoshimoto *et al. *[12] were performed with a *sultr1;3 *mutant in a Wassilewskija ecotype while the present study was performed with mutants in the Columbia ecotype. The use of different ecotypes or perhaps differences in culture media composition or plant age could explain the discrepency. Nevertheless, the current study showed that shoot-to-root sulfate transfer was reduced in the Pi-deficient *sultr1;3 *mutant compared to WT, thus confirming the role of SULTR1;3 in this process. Importantly, the *phr1 *mutant also showed a decrease in shoot-to-root sulfate transfer in Pi-deficient plants relative to WT, revealing the importance of this Pi-signaling transcription factor in the source-sink sulfate distribution. The fact that the reduction in shoot-to-root sulfate transfer observed in the *phr1 *mutant was slightly less compared to the *sultr1;3 *mutant was likely due to the fact that while the *sultr1;3 *mutant completely abolished expression of the protein, some level of *SULTR1;3 *expression still remained in the *phr1 *mutant despite the attenuation in *SULTR1;3 *expression under Pi deficiency. Altogether, these results bring new insights to the regulation and the function of *SULTR1;3 *in Pi-deficient plants and identify PHR1 as an important regulator of *SULTR1;3*.

It is interesting to note that while *SQD1 *and *SQD2*, two genes involved in the replacement of phospholipids by sulfolipids, have been identified as containing a PHR1-binding site in their promoter and are up-regulated by Pi deficiency in a PHR1-dependant manner [29,30], the lipid composition in the *phr1 *mutant indicated that PHR1 had no significant impact on lipid composition in Pi-deficient plants [55]. Thus, while the *phr1 *mutant had previously been shown to be altered in various aspects of Pi metabolism, including Pi allocation between roots and shoots and anthocyanin accumulation, no functional implication of PHR1 in sulfur metabolism has been described [25,31]. In contrast, the present study showed that the implication of PHR1 in the crosstalk between Pi and the sulfate signaling pathway went beyond the regulation of gene expression and that it had an important physiological effect on plant sulfate transport. It is difficult at this point to precisely identify the physiological role of the transport of sulfate from shoots to roots under Pi deficiency. Sulfate is required for the synthesis of numerous compounds that could participate in the Pi-deficiency response, e.g. glutathione levels are known to increase with Pi-deficiency [52]. Pi-deficiency has been associated with an over-accumulation of metals (e.g. iron) and of reactive oxygen species, both of which may trigger the need for additional sulfate for the synthesis of phytochelatin and glutathione [56,57]. It is also possible that the distribution and requirement for sulfate and sulfur-containing compounds may not be homogeneous across the whole root but may be more localized to the cells surrounding the root vascular cylinder. Further studies should thus analyze in more detail the level of sulfate and sulfur-containing compounds in various cell types within the roots.

While the promoter of *SULTR2;1 *contains a sequence homologous to the PHR1-binding site, induction of *SULTR2;1 *by Pi deficiency was not repressed in the *phr1 *mutant. A similar situation was found for the *AtPht1;4 *gene, in which the PHR1 motif was required for gene expression in roots but not for its induction upon Pi starvation in shoots [58]. Furthermore, removal of one of the two P1BS sites present in the *IPS1 *gene abolished its response to Pi deficiency, indicating that the presence of a P1BS element was not sufficient to mediate increased gene expression by Pi deficiency [37]. It is possible that *SULTR2;1 *mRNA abundance is actually more tightly controlled by a different signaling pathway, notably by the action of the transcription factor SLIM1 and miR395, in order to control sulfate transfer in shoots upon Pi starvation [21-23]. In this context, it was recently shown that miR395, a microRNA up-regulated under sulfate deficiency and that targets *SULTR2;1*, was down-regulated under Pi deficiency, thus potentially contributing to *SULTR2;1 *overexpression under Pi deficiency [59]. However, the physiological impact of the down-regulation of miR395 on sulfate metabolism in Pi-deficient plants is not known.

Previous microarray studies showed that a large number of genes were down-regulated by Pi deficiency and use of an inducible PHR1 indicated that transcriptional repression could be indirectly controlled by PHR1 [37]. The increased expression of *SULTR2;1 *and *SULTR3;4 *in shoots of a Pi-deficient *phr1 *mutant relative to Pi-deficient WT plants thus fits with this model of PHR1 playing a key role in both the activation and repression of gene expression. Interestingly, for both *SULTR2;1 *and *SULTR3;4*, increased expression in the *phr1 *mutant was largely confined to the shoot. Distinct expression patterns between shoot and root have been previously noted for *SULTR2;1 *in the context of sulfate deficiency [13,23] and further extended in the present study to Pi deficiency (Figure 2c). For sulfate deficiency, this pattern could be explained by the action of miR395 on *SULTR2;1 *expression in shoots but not in roots because of the non-overlapping tissue-specific expression of miR395 and *SULTR2;1 *in roots [23]. It is thus possible that the distinct response of *SULTR2;1 *and *SULTR3;*4 in roots and shoots of the *phr1 *mutant may also be caused by non-overlapping expression profiles of *PHR1 *and these *SULTR *genes in roots.

## Conclusion

Although the role of the transcription factor PHR1 in Pi homeostasis was previously demonstrated, the present study revealed that PHR1 also plays an important role in sulfate homeostasis in plants grown under Pi-deficient conditions. PHR1 stimulated the expression of the *SULTR1;3 *gene under Pi deficiency, and the significance of this regulation was reflected in a decrease in the shoot-to-root sulfate transfer in the *phr1 *mutant relative to WT. Interestingly, PHR1 also had a repressive role in the expression of the *SULTR2;1 *and *SULTR3;4 *genes in shoots but not in roots. A similar repressive mechanism was recently reported for PSR1, the ortholog of PHR1 in the unicellular alga *Chlamydomonas reinhardtii*. Analysis of the phenotype of the *C. reinhardtii psr1 *mutant grown under Pi-deficiency showed that in addition to altering the normal acclimation to Pi deprivation, the *psr1 *mutant had a de-repressed sulfate deficiency response, leading to overexpression of genes involved in sulfate scavenging and assimilation [41]. Considered together, the results on PSR1 in *C. reinhardtii *and PHR1 in Arabidopsis reveals an unsuspected level of complexity and interconnection in the regulation of sulfate and Pi homeostasis and highlights the evolutionary conservation of the importance of the PSR1/PHR1 transcription factor in these processes.

## Methods

### Plant growth conditions

The *Arabidopsis thaliana *mutants used in all experiments were of Columbia ecotype genetic background. The *phr1 *mutant was kindly obtained from Javier Paz-Ares (CSIC, Madrid) and was previously described [25]. Plants were germinated and grown on agar-solidified media. The complete nutrient medium contained 0.5 mM KNO_3_, 1 mM MgSO_4_, 1 mM KH_2_PO_4_, 0.25 mM Ca(NO_3_)_2_, l00 μM NaFeEDTA, 30 μM H_3_BO_3_, l0 μM MnCl_2_, l μM CuCl_2_, 1 μM ZnCl_2_, 0.1 μM (NH_4_)_6_Mo_7_O_24, _and 50 μM KCl. Sulfate- or Pi-deficient media were made by replacing 1 mM MgSO_4 _or 1 mM KH_2_PO_4 _by 1 mM MgCl_2 _or 1 mM KCl, respectively. Seeds were put on medium-containing plates and left at 4°C in darkness for stratification for 2 days. Plates were then transferred to a growth chamber under the following environmental conditions: light/dark cycle of 8/16 h, light intensity of 250 μmol·m^-2^·s^-1 ^and temperature of 24/20°C. Day one of growth is defined as the first day of exposure of stratified seeds to light.

### Identification of genes containing the P1BS cis-acting element

A custom Python script (available at http://www.unil.ch/dbmv/page12541_en.html) was used to search for the P1BS cis-acting element (GNATATNC) within the 500-bp 5'-upstream regulatory sequences of 33,282 Arabidopsis gene models in the TAIR dataset (TAIR8_upstream_500_20080228) [60].

### Real-Time Quantitative RT-PCR

Total RNA was extracted from frozen shoot and root tissues using the Plant RNeasy extraction kit (Qiagen, http://www.qiagen.com). Any residual genomic DNA was eliminated using a RNAse-free DNAse I (Fermentas, http://www.fermentas.com). Total RNA was quantified with a NanoDrop spectrophotometer (Thermo Scientific, http://www.nanodrop.com). Two micrograms of total RNA were reverse transcribed using the SuperscriptIII RT kit (Promega, http://www.promega.com). Quantitative real-time RT-PCR was performed with a Stratagene Mx3000P apparatus http://www.stratagene.com using SYBR green dye technology (BioRad, http://www.bio-rad.com). The primers used in this work (Additional file 1, Table S1) had an efficiency of amplification ≥ 1.85. PCR reactions were performed in a final volume of 25 μL containing 300 nM each of the forward and reverse primers, 12.5 μL of the SYBR green master mix and 5 μL of a 1:50 cDNA dilution. All PCR reactions were performed in triplicate. For each gene, the relative amount of calculated mRNA was normalized to the level of the control gene ubiquitin mRNA (UBQ10: At4g05320) and expressed as relative values against WT plants grown in complete (+Pi and +sulfate) medium. Reactions were performed in an optical tube (Stratagene) covered with an optical cap (Stratagene). Samples were submitted to 95°C for 15 min, then to 45 cycles of 95°C for 15 s followed by 55°C for 30 s and 72°C for 30 s. Data were analyzed using the MxPro™ software (Stratagene). The specificity of the amplified PCR products and quantification of the relative transcripts levels was performed using the comparative CT method [20,61].

### Identification of the *sultr1;3 *and *sultr2;1 *mutants

The T-DNA insertion mutants *sultr1;3 *(N669442) and *sultr2;1 *(N655235) were from the SALK collection [62] and obtained from the European Arabidopsis Stock Centre http://www.arabidopsis.info. The homozygous mutants were identified and confirmed by PCR using oligonucleotides (see Additional file 1, Table S1, for the sequences).

### Sulfate uptake and transfer measurements

Sulfate transport measurements were performed using whole plants grown *in vitro *for 7 days in medium containing 1 mM PO_4_^2- ^followed by 4 days in medium without PO_4_^2-^. For root-to-shoot measurements, roots of whole plants were placed in a 10 μM Na_2_SO_4 _solution at pH 5.0 in the presence of 1 μCi/mL of the radiotracer ^35^S-Na_2_SO_4 _(PerkinElmer; http://www.perkinelmer.com) for 90 min. Plants were then washed in an ice-cold 5 mM Na_2_SO_4 _solution, and then shoots and roots were harvested separately, blotted with paper towel and the radioactivity measured by scintillation counting. Root-to-shoot sulfate transport was expressed as the percentage of radioactivity located in the shoot over the total amount of radioactivity in the whole plant.

Shoot-to-root sulfate influx measurements were performed using 11-day old plants with similar conditions as described for root measurements, except that the 2 μCi/mL of the radiotracer ^35^S-Na_2_SO_4 _and 0.01% Triton X-100 were deposited on the leaves. Shoot-to-root sulfate transport was expressed as the percentage of radioactivity located in the roots over the total amount of radioactivity in the whole plant.

### Phosphate and sulfate measurements

Anion measurements were performed as described by Rouached *et al. *[20]. Briefly, weighed fresh shoots and roots were ground separately into powder in liquid nitrogen and extracted in water by incubation for 30 min at 70°C. The extract was centrifuged, and the supernatant filtered through a 0.45-μm filter unit. Ion concentration was determined by High-Pressure Ionic Chromatography (ICS-2100 apparatus; Dionex, http://www.dionex.com) using the AS19 anion exchanging column (Dionex) and a KOH gradient. Identification and quantification of Pi and sulfate were performed by comparison of the retention times and peak areas with standards and integrated using the Chromeleon software (Dionex).

## List of abbreviations

Pi: inorganic phosphate; WT: wild type

## Authors' contributions

HR conceived the study; HR, DS and BA performed all experiments; and HR and YP analyzed the data and wrote the paper. All authors discussed the results, read and approved the final manuscript.

## Supplementary Material

Additional file 1 **Table S1: Oligonucleotides used in Q-RT-PCR and mutant identification**. A table describing all oligonucleotides used in Q-RT-PCR and mutant identification in this work Click here for file
