# Supplementary material for: The transcription factor PHR1 plays a key role in the regulation of sulfate shoot-to-root flux upon phosphate starvation in Arabidopsis
Source: BMC Plant Biol. 2011 Jan 24;11:19. doi: 10.1186/1471-2229-11-19 (PMC3036608; doi:10.1186/1471-2229-11-19)
Supplement: Additional file 1 — Table S1: Oligonucleotides used in Q-RT-PCR and mutant identification. A table describing all oligonucleotides used in Q-RT-PCR and mutant identification in this work [file 1471-2229-11-19-S1.PDF]

Table S1. Oligonucleotides used in Q-RT-PCR and mutant identification.

Gene name and AGI number	Forward (F) and reverse (R) primers sequence (5'-3')
SULTR1;3 (At1g22150)	F- 5'TGTCGGCTAGAGCTCATCCTGT3'<br>R- 5'CAATAAGATCGCCGCGAAA3'
SULTR2;1 (At5g10180)	F- 5'TAGTGATCGTTAACCCGAAA3'<br>R- 5'GAGTTACAAACACACCCATC3'
SULTR3;4 (At3g15990)	F- 5'TTGTGCTGGTGAATCCTGTG3'<br>R- 5'GTTTCACAAGAGGCTGCTCC3'
SULTR3;5 (At5g19600)	F- 5'TGATGTTGTCCCATTTCGTG3'<br>R- 5'ACGCATTTTATTAGAACCTTCCCAC3'
SQD1 (At4g33030)	F- 5’ACTCGTCACTAAAGCGGG3’<br>R-5’ CCTGCACAATGTAATATGGA3’
*UBQ*10 (At4g05320)	F- 5'GGCCTTGTATAATCCCTGATGAATAAG3'<br>R- 5'AAAGAGATAACAGGAACGGAAACATAGT3’
Tubulin (At1g04820)	F- 5’AAATTAGGGTTTCTACTGAGAGAAG3’<br>R- 5’ACGAATATTTTACAGGATTTAAACA3’
sultr1;3 T-DNA mutant	F- 5’ATGACGGAGAGATTTCCCCGGTG3’<br>R- 5’TAGTGAATTCTCAGACCTCGTCGGAC3’
sultr2;1 T-DNA mutant	F- 5’GGGGACATCAAGAGAGATAGC3’<br>R- 5’TTACGGTTAGAGCAACAATGG3’
